# Supplementary material for: Potential of organizing unmarried adolescent girls and young women into self-help groups for a better transition to adulthood: Findings from a cross-sectional study in India
Source: PLoS One. 2021 Mar 18;16(3):e0248719. doi: 10.1371/journal.pone.0248719 (PMC7971707; doi:10.1371/journal.pone.0248719)
Supplement: Attachment — Submitted filename: Response to Reviewers.docx [file pone.0248719.s001.docx]

*January 18, 2021*

**“Potential of organizing unmarried adolescent girls and young women into self-help groups for a better transition to adulthood: Findings from a cross-sectional study in India” (Ref No.** **PONE-D-20-29446R1)**

**Response to the reviewer and editor’s comments**

| **S. N.** | **Comments** | **Author’s responses** |
| --- | --- | --- |
|  | **Editor’s comments** |  |
|  | The academic Editor served as the second reviewer on this manuscript. The editor agreed with the first reviewer. The authors also need to add a clear set of actionable recommendations linked to the key findings. | We reviewed the manuscript and added a set of actionable recommendations in the last para of the discussion (line number 350-368) as;  “The study noted that AGWYs aspired for higher education – both those who were currently in school or college and those who had dropped out of school. Education is critical for the overall development of an individual. The schools and colleges provide space and opportunities for AGYWs to achieve their educational aspirations. Therefore, programs addressing AGYWs lives must address the issues around drop out of girls from school and colleges. This study found that YWSHG members compared to non-members had greater individual agency, thus demonstrating that YWSHGs are safe spaces for AGYWs in rural settings to channel their potential and energies. Such groups provide them space to interact and a safety net for taking collective action. The finding that AGYWs members were twice more likely to make decisions on matters that concern them indicate that YWSHGs help in transform gender attitudes and norms which are critical for girls’ future lives. The findings thus demonstrate that YWSHGs have the potential to empower young women to be agents to change and transform future generations. Therefore, it is important to continue the mobilization of AGYWs particularly in rural settings in India. Now that under the National Rural Livelihood Mission, the Government of India is making a deliberate attempt to improve the livelihood opportunities and empower women through SHGs, it may be equally important to invest and support in developing and designing or replicating such models of organizing AGYWs into YWSHGs, to implement interventions that improve the skills and lives of adolescents and young women.” |
| 1 | Please ensure that your manuscript meets PLOS ONE's style requirements, including those for file naming. The PLOS ONE style templates can be found at  https://journals.plos.org/plosone/s/file?id=wjVg/PLOSOne_formatting_sample_main_body.pdf and  https://journals.plos.org/plosone/s/file?id=ba62/PLOSOne_formatting_sample_title_authors_affiliations.pdf | We have thoroughly reviewed the manuscript and ensured formatting according to PLOS ONE’s style requirements |
| 2 | Thank you for stating the following in the Competing Interests section:  "The authors have declared that no competing interests exist"  We note that one or more of the authors are employed by a commercial company: name of commercial company. | One of the authors (Arima Singh) was involved with the study and manuscript writing while she was associated with the organization that was working with YWSHGs. Currently, she is employed by IPE Global Pvt. Ltd., an international development consulting company that did not have any role either in conduct of the study or writing of the manuscript.  We have revised the ‘funding statement’ and ‘competing interests’ statement’ to reflect this (please see response in point number 2.1 and 2.2). |
| 2.1 | Please provide an amended Funding Statement declaring this commercial affiliation, as well as a statement regarding the Role of Funders in your study. If the funding organization did not play a role in the study design, data collection and analysis, decision to publish, or preparation of the manuscript and only provided financial support in the form of authors' salaries and/or research materials, please review your statements relating to the author contributions, and ensure you have specifically and accurately indicated the role(s) that these authors had in your study. You can update author roles in the Author Contributions section of the online submission form.  Please also include the following statement within your amended Funding Statement.  “The funder provided support in the form of salaries for authors [insert relevant initials], but did not have any additional role in the study design, data collection and analysis, decision to publish, or preparation of the manuscript. The specific roles of these authors are articulated in the ‘author contributions’ section.”  If your commercial affiliation did play a role in your study, please state and explain this role within your updated Funding Statement. | We have revised the funding statement and included  “The Population Council was funded for this study by the Bill & Melinda Gates Foundation (Grant OPP1141832). The funder provided support in the form of salaries for authors [JA, AH, KA], but did not have any additional role in the study design, data collection and analysis, decision to publish, or preparation of the manuscript. The specific roles of these authors are articulated in the ‘author contributions’ section. At the time of conduct of the study and manuscript development, AS was employed by the organization that was working with YWSHGs. Currently, AS is employed by a commercial organization that did not have any role in this study.” |
| 2.2 | Please also provide an updated Competing Interests Statement declaring this commercial affiliation along with any other relevant declarations relating to employment, consultancy, patents, products in development, or marketed products, etc.  Within your Competing Interests Statement, please confirm that this commercial affiliation does not alter your adherence to all PLOS ONE policies on sharing data and materials by including the following statement: "This does not alter our adherence to PLOS ONE policies on sharing data and materials.” (as detailed online in our guide for authors <http://journals.plos.org/plosone/s/competing-interests>) . If this adherence statement is not accurate and there are restrictions on sharing of data and/or materials, please state these. Please note that we cannot proceed with consideration of your article until this information has been declared.  Please include both an updated Funding Statement and Competing Interests Statement in your cover letter. We will change the online submission form on your behalf.  Please know it is PLOS ONE policy for corresponding authors to declare, on behalf of all authors, all potential competing interests for the purposes of transparency. PLOS defines a competing interest as anything that interferes with, or could reasonably be perceived as interfering with, the full and objective presentation, peer review, editorial decision-making, or publication of research or non-research articles submitted to one of the journals. Competing interests can be financial or non-financial, professional, or personal. Competing interests can arise in relationship to an organization or another person. Please follow this link to our website for more details on competing interests: [http://journals.plos.org/plosone/s/competing-interests](about:blank) | Thank you for raising the concern. We carefully reviewed the details on competing interests through the link you provided ([http://journals.plos.org/plosone/s/competing-interests](about:blank)). We have revised the Competing Interests Statement as below:  “The authors declare no individual or organizational competing interest exist. Commercial affiliation of one author (AS) does not alter our adherence to PLOS ONE policies on sharing data and materials.”  Based on your advice, we have included both an updated Funding Statement and Competing Interests Statement in the revised cover letter. |
| 3 | We note that you have stated that you will provide repository information for your data at acceptance. Should your manuscript be accepted for publication, we will hold it until you provide the relevant accession numbers or DOIs necessary to access your data. If you wish to make changes to your Data Availability statement, please describe these changes in your cover letter and we will update your Data Availability statement to reflect the information you provide. | Please find below the data repository information (DOIs) to access the data, study tool and a readme file that includes description about the data.  <https://doi.org/10.7910/DVN/NQYBPM> |
|  | **Reviewer’s Comments** |  |
| 1 | Is the manuscript technically sound, and do the data support the conclusions?  The manuscript must describe a technically sound piece of scientific research with data that supports the conclusions. Experiments must have been conducted rigorously, with appropriate controls, replication, and sample sizes. The conclusions must be drawn appropriately based on the data presented.  Reviewer #1: Yes | Thank you for your appreciation |
| 2 | Has the statistical analysis been performed appropriately and rigorously?  Reviewer #1: I Don't Know | We have applied appropriate statistical techniques to examine the study objectives. |
| 3 | Have the authors made all data underlying the findings in their manuscript fully available?  The PLOS Data policy requires authors to make all data underlying the findings described in their manuscript fully available without restriction, with rare exception (please refer to the Data Availability Statement in the manuscript PDF file). The data should be provided as part of the manuscript or its supporting information, or deposited to a public repository. For example, in addition to summary statistics, the data points behind means, medians and variance measures should be available. If there are restrictions on publicly sharing data—e.g. participant privacy or use of data from a third party—those must be specified.  Reviewer #1: No | Please find below the data repository information (DOIs) to access the data, study tool and a readme file that includes description about the data.  <https://doi.org/10.7910/DVN/NQYBPM> |
| 4 | Is the manuscript presented in an intelligible fashion and written in standard English?  PLOS ONE does not copyedit accepted manuscripts, so the language in submitted articles must be clear, correct, and unambiguous. Any typographical or grammatical errors should be corrected at revision, so please note any specific errors here.  Reviewer #1: No | We have revised the manuscript and done copyediting to avoid any typographical or grammatical errors. |
| 5 | **Review Comments to the Author**  Please use the space provided to explain your answers to the questions above. You may also include additional comments for the author, including concerns about dual publication, research ethics, or publication ethics. (Please upload your review as an attachment if it exceeds 20,000 characters) |  |
| 5.1 | Reviewer #1: - The title is vague since the focus is not evident until you read the abstract that the authors assess transition to adulthood. I would strongly recommend adding transition to adulthood to title to let focus be known. | We have reassessed the title. In light of the outcome measures we have used in the paper, we have revised the title of the manuscript as “Potential of organizing unmarried adolescent girls and young women into self-help groups for a better transition to adulthood: Findings from a cross-sectional study in India. |
| 5.2 | -Copyediting and proofreading the entire manuscript is strongly recommended | We have thoroughly reviewed the manuscript and did the copyediting to avoid spelling and grammatical errors. |
| 5.3 | -Authors state that “The Government of India and the State government have launched several schemes and programs for adolescents girls to provide opportunities to improve their health and well-being [19]. But the services have not reached the target group adequately for many reasons including insufficient awareness and lack of knowledge among adolescents.” Not sure how insufficient awareness and lack of knowledge among adolescents are different reasons. What are the other “many reasons?” | We have revised the statement and expanded “many reasons”. The revised text in the manuscript (line number 61-67) is as follows:  “The Government of India and the State government have launched several schemes and programs for adolescents’ girls to provide opportunities to improve their health and well-being [18]. But many services have not reached the target group adequately for many reasons including lack of (correct) information among adolescents and /or their parents about schemes, absence of proper guidance to access schemes, challenges in accessing the services due to poor implementation and insufficient services from system [9]”. |
| 5.4 | - Need to explicitly state measures and scales used for other researchers to replicate. Would strongly advise to add data collection instruments to appendix to understand context of scales and measures to see why variables were measured the way they were (e.g. standard of living, exposure to mass media, etc.). | We have elaborated the measures about the scales used in the paper and given proper citation to refer to the original sources from where we adopted the questions/scales. The instrument used for the present study is available at  <https://doi.org/10.7910/DVN/NQYBPM>  We have explained the construction of standard of living index (Line no. 202-206) and exposure to mass media (Line no.201). The revised text is as below  SLI: “A proxy variable of standard of living index (SLI) was computed to denote a household’s economic condition and was used in the analysis. The SLI computation was based on an additive index using four variables available in the dataset – main source of drinking water, main source of lighting for the household, main fuel used for cooking in the household and type of house.”  Mass media exposure: “either reads newspaper or listen to radio or watches television”. |
| 5.5 | - Information regarding the caste system would be helpful to provide context for readers. | We have added information on caste system. The text is added in Line no. 122-124 appears as:  “Historically, the SCs and STs have been socially and economically marginalized segments in Indian society and the proportion of such a population within a geographic location influences many developmental parameters.” |
| 5.6 | - Would suggest in the discussion when authors state “These findings were similar to the findings of the youth survey [12],” authors should give context about the youth survey referenced since it currently reads as a youth survey that was part of the authors' study even though that is not the case. | The context has been added to the reference of the youth survey. The revised text in Line no. 303-306 appears as:  “Restricted mobility, and limited agency among unmarried AGYW was also reported in “Youth in India” study that was conducted in six states of India to assess the situation and needs of the adolescent and young population [12]”. |
| 5.7 | - Great job to the authors for combining multiple programmatic approaches to promote individual agency to incorporate nutrition, menstrual health and hygiene. | Thank you for the appreciation. |
